# Ethical challenges in global health-related stigma research

**DOI:** 10.1186/s12916-019-1317-6

**Published:** 2019-04-29

**Authors:** Joseph Millum, Megan Campbell, Florencia Luna, Arianne Malekzadeh, Quarraisha Abdool Karim

**Affiliations:** 1 Clinical Center Department of Bioethics, National Institutes of Health, 10/1C118, 10 Center Drive, Bethesda, MD 20892 USA; 2 Fogarty International Center, National Institutes of Health, 31 Center Dr, Bethesda, MD 20892 USA; 3 Department of Psychiatry and Mental Health, University of Cape Town, J-Block, Groote Schuur Hospital, Observatory, Cape Town, South Africa; 4 CONICET, Programa de Bioética, FLACSO Argentina, Tucumán 1966, C1050AAN Caba, Argentina; 5 Centre for the AIDS Program of Research in South Africa, Doris Duke Medical Research Institute, Nelson R Mandela School of Medicine, University of KwaZulu-Natal, Congella, 4013 South Africa

**Keywords:** Stigma, Ethics, Global health, Research ethics, Vulnerability, De-normalization, Research risks, Confidentiality, Privacy

## Abstract

**Background:**

It is critically important to conduct research on stigmatized conditions, to include marginalized groups that experience stigma, and to develop interventions to reduce stigma. However, such research is ethically challenging. Though superficial reference is frequently made to these widely acknowledged challenges, few publications have focused on ethical issues in research on stigmatized groups or conditions. In fact, a brief literature review found only two such publications.

**Main text:**

At a recent Science of Stigma Reduction workshop comprising 60 stigma researchers from the USA and low and middle-income countries, the need for more robust and critical discussion of the ethics of the research was highlighted. In this paper we describe, illustrate through cases, and critically examine key ethical challenges that are more likely to arise because a research study focuses on health-related stigma or involves stigmatized groups or conditions. We examine the ethics of this research from two perspectives. First, through the lens of overprotection, where we discuss how the perception of stigma can impede ethical research, disrespect research participants, and narrow the research questions. Second, through the lens of research risks, where we consider how research with stigmatized populations can unintentionally result in harms. Research-related harms to participants include potential breaches of confidentiality and the exacerbation of stigma. Potential harms also extend to third parties, including families and populations who may be affected by the dissemination of research results.

**Conclusions:**

Research with stigmatized populations and on stigmatized conditions should not be impeded by unnecessary or inappropriate protective measures. Nevertheless, it may entail different and greater risks than other health research. Investigators and research ethics committees must be particularly attentive to these risks and how to manage them.

## Background

Stigma is common globally for multiple and diverse reasons. Patients may be stigmatized because they have diseases, such as HIV/AIDS, leprosy, lung cancer, epilepsy, or schizophrenia, or for characteristics or behaviors that are regarded as undesirable or “socially deviant”, e.g. because they smoke, inject drugs, are obese, are sexually or gender non-conforming, or drink alcohol during pregnancy. The nature of stigma varies, but stigma and its effects are found everywhere.

It is critically important to conduct research on stigmatized conditions, to include marginalized groups that experience stigma, and to develop interventions to reduce stigma. However, such research is operationally and ethically challenging. As we illustrate in this paper, individuals who experience stigma may be hard to recruit, participants may be at higher risk of certain harms, and the results of research could lead to further marginalization or other negative effects on at-risk communities.

In this paper, we describe, illustrate through cases, and critically examine key ethical challenges that are more likely to arise because a research study focuses on health-related stigma or involves stigmatized groups or conditions. Our goals are threefold: (1) to help researchers, research ethics committees (RECs), and other stakeholders to appreciate the range of ethical challenges that research with stigmatized groups or on stigmatized conditions presents; (2) to make recommendations regarding those challenges, where that is possible given existing resources; and (3) to identify areas where the challenges merit further work.

We approach this topic with an understanding that there are commonalities across populations, conditions, and types of research. The stigma that makes research with HIV-positive adolescents ethically challenging, for example, has some features in common with the stigma that poses difficulties for responsible research on the genetic inheritance of schizophrenia. We hope lessons learned in one area, suitably adjusted in the light of contextual differences, can help researchers facing similar challenges in another.

In this paper, stigma is understood as a socially constructed phenomenon that occurs when members of a group experience status loss or discrimination on the basis of some shared characteristic that is deemed undesirable by a dominant group [1]. Its effects can occur through attitudes and beliefs internalized by stigmatized individuals (self-stigma), through overt discrimination by others (experienced or enacted stigma), and through the fear of such discrimination (felt stigma). This broad definition is intended to be maximally inclusive of situations in which researchers encounter the challenges we discuss.

In June 2017, the Fogarty International Center of the National Institutes of Health hosted a three-day workshop on ‘The Science of Stigma Reduction: New Directions for Research to Improve Health’. Workshop attendees included approximately 60 researchers from the USA and low and middle-income countries whose work addresses stigma related to different disease areas and populations. During one session of the workshop, participants discussed the ethical challenges they faced when conducting research with stigmatized groups or on stigmatized conditions. Multiple participants noted a lack of guidance specific to these challenges.

We conducted a literature review using two electronic database sources, Google Scholar and PubMed. We searched for all papers, in English, containing the terms “stigma” and “ethics” anywhere in the article. We manually reviewed the titles and abstracts of the resulting publications to determine their relevance to health-related stigma and to research ethics.

Most of the ethics literature relating to stigma focuses on public health interventions, such as anti-tobacco or obesity campaigns. We identified only two papers that focused on the ethics of research on stigma or involving stigmatized groups [2, 3]. Researchers and research ethicists are clearly cognizant of ethical challenges arising from stigma research, because it is mentioned in multiple places in prominent guidance documents [4, 5]. However, the issue is typically diluted or subsumed under broader categories. For example, many populations at risk for stigma are considered to be ‘vulnerable’, but this label is also applied to populations that are not stigmatized, such as children. At other times, stigma is raised in the context of a specific disease or type of research (e.g. HIV/AIDS or genetic research), where valuable insights into how to conduct research ethically have been developed, but the siloed nature of research means that these insights are not always communicated to researchers working in other topic areas for which stigma is an issue.

The discussion at the workshop, subsequent follow-up with participants, and the literature review, highlighted multiple ethical issues. From these, we identified a subset that appear more likely to arise because a research study focuses on health-related stigma or involves stigmatized groups or conditions. For each, we selected a case study – one that had either been contributed by a workshop participant or described in the literature – that illustrates the ethical issue, and applied ethical principles – as articulated elsewhere in research ethics – to analyze it.

## Ethical challenges in stigma research

### Overprotection

Because they are often severe and hard to treat or prevent, there is frequently a particular need to conduct research into stigmatized conditions. Likewise, people who experience stigma are typically marginalized and in greater need of assistance than those with a socially accepted status. More research is also needed into interventions to reduce stigmatization itself, given its negative effects on health and wellbeing. Despite the need, substantial barriers impede this research.

One key barrier is the link between stigma and perceived vulnerability. In research ethics, ‘vulnerability’ has traditionally been used to label populations that are thought to be at greater risk of harm or some other wrong [6], including children, people in subordinate positions or who are poor, ethnic and racial minorities, and the mentally ill [7]. Protections for vulnerable populations are sometimes enshrined in laws; even outside of regulatory restrictions, funders and RECs are often reluctant to allow research with populations regarded as vulnerable. This traditional conceptualization of vulnerability may lead to two forms of overprotection: overprotection through exclusion from research, and through providing inappropriate protections in research. The first two cases illustrate these problems. The third case illustrates a different form of overprotection, which arises because of the presumption that stigma is invariably harmful and counterproductive. This last case raises the question of whether there are contexts in which health researchers should investigate beneficial effects of de-normalizing certain behaviors.

#### Case 1. Adolescents excluded from PrEP studies

People infected with HIV, children living with family members with HIV, and children who are orphaned because of HIV are frequently stigmatized [8]. This stigma reduces health-seeking behaviors and is a major obstacle to treatment and prevention efforts. Adolescents represent a large proportion of people living with HIV globally: one-third of all new HIV infections in 2016 occurred in adolescents aged 15–19 years [9]. In sub-Saharan Africa, gendered power disparities, gender-based violence, and the consequent inability to negotiate safer sex practices, exacerbate the vulnerability of adolescent girls to HIV. Adolescent sexual activity is also frequently stigmatized. While antiretroviral PrEP offers a female-centered approach, with demonstrated prevention potential in adherent women aged 18 or over, adolescents below 18 are inadequately represented in PrEP trials [10]. Stringent ethical–legal guidelines and RECs in South Africa require parent/guardian consent to participate in clinical trials for everyone under the age of 18 because they are considered to be vulnerable. Moreover, in the worst hit provinces, one-fifth of children in many communities have lost parents (often to AIDS), and one-third do not live with either biological parent [11]. There is often no formally assigned guardianship, especially in rural communities. The adolescent girls who are most at risk are those most affected by the negative effects of secondary HIV stigma (that is, stigma attached to those who are associated with individuals stigmatized because of their HIV status). Yet, tragically, these are the same girls whom it is most difficult to enroll into studies into ways to prevent HIV. The cause of their stigmatization – coming from HIV-affected families – not only deters them from engaging with HIV researchers and clinicians, but also makes it particularly hard to get consent from a parent to enroll them into clinical trials.

#### Case 2. Schizophrenia patients and consent capacity

The Genomics of Schizophrenia in South African Xhosa People study was a psychiatric genomics study that examined gene mutations in Xhosa people with schizophrenia and unaffected controls [12]. As in many other countries, schizophrenia is heavily stigmatized in South Africa. The REC that approved the study required screening for decisional capacity for participants with schizophrenia. The underlying assumption was that schizophrenia was liable to impede the capability to make informed decisions, and schizophrenia patients needed particular protection against inappropriate enrollment. Interestingly, use of an informed consent screening tool to evaluate the quality of understanding of the research study demonstrated that, while many individuals with schizophrenia struggled to understand certain elements of the study during recruitment, so did some of the unaffected controls [13]. Rather than simply excluding people with schizophrenia who did not exhibit sufficient understanding, the researchers developed an iterative learning process to use with all potential participants. Using a brief screening tool, they assessed understanding of different research study elements, such as the study’s aim, risks, and benefits. This allowed them to revisit and better explain elements that were hard to grasp, improving participant understanding. The iterative process, while more time consuming, demonstrated large improvements in understanding in both study groups [13].

#### Case 3. Public health interventions to prevent fetal alcohol syndrome

Alcohol consumption during pregnancy is associated with fetal alcohol spectrum disorders (FASD), which encompass a range of mental, physical and neurodevelopmental deficits in infants, including fetal alcohol syndrome (FAS) [14]. Global prevalence of alcohol consumption during pregnancy is estimated at 9.8%, and an estimated 119,000 babies are born with FAS each year [15]. New behavioral interventions to reduce alcohol consumption before and during pregnancy are urgently needed, including in countries where contact with a clinician before or early in pregnancy is not routine for many women. One plausible basis for public health interventions is to attempt to de-normalize drinking during pregnancy through, for example, targeted warning labels on alcoholic drinks or advertising campaigns. The de-normalization of smoking is widely thought to have contributed to dramatic declines in tobacco use in many high-income countries. However, there are concerns that public health campaigns that encourage people to regard drinking during pregnancy as socially unacceptable would also exacerbate the stigmatization of people with FASD and their parents. Should researchers develop and study such de-normalizing interventions?

### Critical discussion

Overprotection, even if well-intentioned, can have negative consequences. In Case 1, adolescents are prevented from participating in research that would address their urgent need for safer, more effective HIV prevention. A population in great need of an effective intervention may therefore be substantially delayed in receiving it. In Case 2, people who might make a meaningful contribution to psychiatric genomics research could have been excluded, and unaffected controls could have been recruited without proper understanding of the study. RECs typically voice concern about the capacity of people with severe mental illness to give informed consent. It then becomes the responsibility of the research team to demonstrate adequate consent. However, in being overly cautious of protecting against the exploitation of a stigmatized group, researchers may not pay sufficient attention to the particular needs of that group to promote their inclusion, or recognize the complexities of the research study elements that may impact on understanding for everyone.

#### Vulnerability

Overprotection is closely related to labeling a population as ‘vulnerable’. Once a stigmatized population is classified as vulnerable, protection is required, the default of which is frequently exclusion from research. The traditional or subpopulation approach to vulnerability, according to which entire populations are classified as vulnerable, also has a stereotyping effect because the label ‘vulnerable’ cannot be easily removed and can thereby exacerbate stigmatization [16]. RECs tend to use the concept of vulnerability in this traditional way.

An alternative view of vulnerability may help researchers working with stigmatized groups to address this problem. Instead of the subpopulation approach, we can consider vulnerability in a layered way [17]. This concept of vulnerability is relational: if the context changes, the person may no longer be vulnerable in that way [4]. Some layers may be related to problems with informed consent, others to violations of human rights or social circumstances, and they may overlap or compound. In this way, the layered view of vulnerability shares features with the concept of intersectionality. Intersectionality refers to the way that an individual may belong to multiple groups, each of which faces discrimination, and the forms of discrimination experienced by someone with this overlapping membership may not be reducible to the discrimination experienced by individuals who belong to just one of the groups [18].

For example, a woman, in herself, is not vulnerable, but a woman living in a country that is intolerant of reproductive rights acquires a layer of vulnerability. In turn, an educated and well-off woman in that same country might overcome some of the consequences of such intolerance, while a poor woman acquires another layer of vulnerability. Moreover, an illiterate, poor woman acquires still another layer. On this view, vulnerability is not a binary category: the metaphor of layers gives flexibility to the concept.

The layered view of vulnerability can help to evaluate proposed research projects involving stigmatized groups. First, researchers should identify potential layers of vulnerability. Second, they should consider strategies for managing each layer in ways that seek to safely include – as oppose to exclude – potential participants [19]. RECs and researchers should design tools to empower research participants – helping them to make their own choices and pursue their own goals – as well as providing adequate safeguards and protections. As DuBois et al. recommend, “Offer as many protections as necessary and as few as possible [20].”

For example, in Case 1, age of consent can be considered as a layer of vulnerability. In this case, the researchers seeking to include adolescent girls sought to manage the vulnerability by using community engagement mechanisms to promote appropriate consent and protection. Members of the local community, including adolescent girls, were asked to advise on how to involve this group in research in ways that retained trust in the research enterprise, encouraged them to seek care, and reduced the risks of exacerbating HIV-related stigma. The solution proposed and presented to the REC involved having a community adult proxy to serve as guardian for adolescent participants who lacked a formal guardian, as well as comprehension tests for the participants themselves. The approach of engaging the community also drew attention to the existence of child-headed households and catalyzed community support for them. For Case 2, if we consider decisional capacity as a layer of vulnerability that affects participants to varying degrees, the focus during recruitment becomes more about how to assist understanding of the research study than about who to exclude. In this way, we guard against overcautious exclusion and perpetuating negative stereotypes and stigma.

#### De-normalization

A final issue relating to overprotection concerns the research questions that are asked about stigma, as Case 3 illustrates. Stigma and stigmatization have been the target of sharp critiques from public health advocates and social scientists for decades. Stigmatization, it is argued, threatens populations by driving its targets to the margins of society and reinforcing negative stereotypes. Stigmatization has therefore been denounced as morally repugnant, as unjust, and as a violation of human rights [21]. In the context of FASD, multiple commentators have raised concerns that behavioral interventions to reduce alcohol consumption during pregnancy might increase the felt or enacted stigma of people with FASD and their parents [22]. Moreover, they claim, stigmatizing alcohol use in pregnancy might be counterproductive by discouraging women from admitting alcohol use or seeking prenatal care [23, 24].

Evidence suggests that alcohol warning labels increase awareness [25], and graphic warning labels with pictures are effective in reducing tobacco use [26]. However, there is a paucity of data regarding the net benefits or harms of interventions that might reduce drinking during pregnancy through such public health campaigns. Public health campaigns that aimed to de-normalize certain forms of previously socially acceptable behavior, such as smoking, have had some success in reducing the harmful behaviors they target [27, 28]. They may also have indirect negative consequences, such as when lung cancer patients are stigmatized because they are seen as responsible for their disease [29]. The line between de-normalization that leads individuals engaging in unhealthy behaviors to regard those behaviors as no longer socially acceptable, and the status loss and discrimination associated with stigma, is difficult to draw.

In summary, FASD is a huge health problem, we lack proven effective interventions, and there are several potential de-normalizing interventions that might have beneficial and potentially negative effects. In such circumstances, it seems prudent to conduct research to find out what the actual effects would be. We see this as a challenge to both critics and proponents of public health campaigns that could create or exacerbate stigma. Those who think we should not even consider research to test de-normalizing interventions need to provide good reasons for their case; for example, high-quality evidence that the strategy would be ineffective or harmful on balance. Speculative claims about harm are insufficient. On the other hand, those who think we should consider interventions that risk stigmatizing pregnant drinkers should develop de-normalization interventions intended to minimize harms and maximize benefits. Such interventions need rigorous study so that they will be adopted (or not) on the basis of data regarding their effects.

### Research risks

Stigma poses additional risks to research participants. Fear of stigmatization and discrimination affects individuals’ willingness to leave their homes, engage publicly, and obtain healthcare services. As a result, some stigmatized groups are difficult to access for healthcare and research. One key challenge, then, is how researchers should interact with the most severely stigmatized populations, when the stigma is itself a powerful barrier, and association with the research may lead to substantial harms. Patients with stigmatized conditions may also be at greater risk of harms from the research procedures themselves, where they risk perpetuating stigma or re-traumatizing participants. Finally, research risks are not limited to research participants, but can also affect their families and communities. The following cases illustrate these points.

#### Case 4. African immigrants living with HIV (unpublished observations, Deepa Rao)

African immigrant communities in the Seattle area tend to be small and tightknit, with little anonymity. Consequently, many community members living with HIV fear inadvertent disclosure of their HIV status. They are reluctant to be seen in HIV care-related settings, especially by other community members. They avoid tangible association with the disease, including participation in HIV-related research, and appear to be unfamiliar with clinical research in general. They have significant concerns about having their data collected and personal information recorded, especially related to HIV. A related fear is that disclosure of their HIV status may jeopardize their immigration status. Many African immigrants also struggle with language and literacy barriers, making the conveying of sensitive information and reassurances challenging.

#### Case 5. Women with epilepsy (unpublished observations, Gretchen Birbeck)

Women with epilepsy are stigmatized in many settings. Focus group discussions with women with epilepsy in communities in Zambia revealed traumatic stories of spousal abandonment in the days, months, and sometimes years after their condition developed or became public knowledge. Hearing about this was frightening for other women in the focus groups who had not (yet?) been abandoned by their spouses. In the same study, based on requests from local support groups, hats and t-shirts making reference to bringing epilepsy “out of the shadows” were distributed to those living with the condition. On bringing these items home, some women were physically and verbally abused by their family members who feared that their condition would result in the whole family being stigmatized.

#### Case 6. The Maori and a “gene for” aggression

Monoamine oxidases (MAOs) are a family of enzymes that break down neurotransmitters. In the early 2000s, studies identified a correlation between an MAO-A gene variant and anti-social behavior in Caucasian men who were abused or neglected in childhood [30]. It was described in a 2004 *Science* report as a “warrior” gene [31]. In 2006, researchers from New Zealand’s Institute for Environmental Science and Research presented the results of their research on the MAO-A gene variant in a Maori (indigenous New Zealand) population under the title, ‘Tracking the evolutionary history of the warrior gene in the South Pacific.’ One of the researchers involved commented: “Obviously, this means they are going to be more aggressive and violent, and more likely to get involved in risk-taking behavior like gambling… It is controversial because it has implications suggesting links with criminality among Maori people [32].” Headlines around the world quickly reflected this claim, which played into existing stereotypes about the Maori, despite the scientific basis for the behavioral claims being debunked [30].

### Critical discussion

As these cases illustrate, research with stigmatized groups or on stigmatized conditions may pose additional risks that would not typically arise in research on populations who were not stigmatized. Two types of risk warrant particular attention: risks related to privacy and confidentiality, and risks to non-research participants.

#### Privacy & confidentiality

Researchers have a duty to preserve the privacy and confidentiality of all participants. This duty is frequently more weighty and broader in scope for research with stigmatized groups or on stigmatized conditions [33]. It is weightier because the negative consequences of breaches are often higher; for example, knowledge, or even suspicion of a diagnosis of leprosy might lead to someone being socially ostracized. There may even be legal consequences; for example, people who inject drugs face stigma, but are also likely to be breaking the law. It is broader insofar as the scope of the information that needs to be kept private to protect participants may be greater than in other research contexts. For example, simply being seen visiting a clinic that is known to conduct HIV-related research may imply to others that a participant has HIV.

Where the risk to participants of being identified from research data is high, full anonymization should be considered and implemented as soon as the scientific goals of the research allow. Whether such anonymization is possible or desirable will depend on the nature of the research. For example, biobanks may de-identify specimens, but modern genetics techniques mean that it is no longer possible to guarantee against re-identification [34]. Some funding organizations and journals require researchers to make their data publicly available. Caution should be exercised in how this is done for research where re-identification of participants would put them at considerable risk [4]. For research participants who engage in illegal activities, additional protections are sometimes available. For example, in the USA, the National Institutes of Health issues Certificates of Confidentiality that protect researchers from being compelled to release identifying information about participants during legal proceedings [35].

In many cases, the risk of breach of confidentiality does not come from the data that has been collected, but from the interaction of participants with researchers, such as when the researchers are known to be studying a stigmatized condition or population. Depending on the population or site, this risk can sometimes be mitigated. For example, Sugarman et al. describe a process for developing site-specific participant safety plans, which they used in a multinational HIV prevention study with people who inject drugs [36]. The plans were based on local legal and policy assessments, and semi-structured interviews with key stakeholders (such as people who inject drugs, clinicians who treat drug use or HIV, law enforcement officials, and policy experts). Distinctive features of the plans included describing the study as being about HIV prevention rather than about drug users or people with HIV/AIDS, conducting the study at sites where several medical services were provided, and training staff on confidentiality and stigma reduction. In another HIV study, this time of group cognitive-behavioral therapy to reduce alcohol use among HIV-infected outpatients in Western Kenya, the investigators describe several changes to their practices to protect participants from breaches of confidentiality. These included disregarding the cultural expectation that consent from women would be obtained only after permission was granted by the male head of household, and ceasing to offer free taxis to the study site once it was noted that this called attention to participants [37].

#### Risks to third parties

The core value of research cannot be realized unless its results are publicized. However, as Case 6 shows, it matters which results are disseminated and how. There, the framing of the results encouraged a simplistic understanding of the underlying science that played into existing negative stereotypes, and was, predictably, picked up by the media. Achkar and Macklin, who describe the pros and cons of reporting findings from research on undocumented immigrants in the USA, raised parallel concerns about possible uptake [2]. The results seemed to imply that undocumented immigrants were more likely to transmit tuberculosis than documented immigrants or US-born persons. Would publishing these findings exacerbate the stigmatization of undocumented immigrants?

How the results of research will be disseminated, and how they might be used or misused, should be considered in the planning stage of a research study. Researchers should plan, for example, how to convey their results to policy-makers, and how to minimize the risks of misinterpretation. When planning to study a population at risk for stigma, researchers should ask themselves: is this research project likely to lead to knowledge that benefits this population? If not, then they probably should not be asking those research questions, or should redesign the study. In their discussion of the study of immigrants and tuberculosis, Achkar and Macklin are careful to emphasize that publishing the data relating to undocumented immigrants had a plausible connection to public health interventions likely to benefit those immigrants.

Some potential ‘group harms’ [38] from stigma research could be addressed through appropriate community engagement. Community engagement provides an opportunity to inform community members about the research, and to get permission for the research from community representatives (over and above the consent of individual research participants). Engaging with communities is not just about getting permission; it is about understanding the perspectives of people who may be affected, the risks they perceive, and how health-related stigma is perpetuated in that specific context. This is critical when outside researchers attempt to work with stigmatized populations; for example, high-income country researchers working on stigma in low or middle-income countries. At its best, community engagement means involving stigmatized groups in the research process, and empowering them through ownership of the research [39].

There is extensive literature on how to involve communities in planning and conducting research, including non-traditional communities, such as patient groups [40, 41]. Researchers might draw on the existing work regarding community engagement and community advisory boards [42]. Best practices for research with indigenous peoples may also provide helpful lessons for other research with marginalized populations, including those who experience stigma [5, 43]. It is worth noting, however, that this is another area in which the distinctive nature of stigma sometimes supports a different approach. For example, for research on a non-stigmatized condition, the goals of community engagement might be best realized by involving community members in data collection. By contrast, research with HIV and tuberculosis patients suggests that these patients may prefer to interact with non-community members to reduce the risks of a breach of confidentiality [44]. Again, careful engagement with community members, especially those drawn from affected populations, can help to identify such risks.

Another third party that can be affected by research without being enrolled is the participant’s family. In Tekola et al.’s discussion of informed consent for genetic research on podoconiosis in southern Ethiopia, they note: “Patients were concerned that the research might publicize podoconiosis as a familial condition and would aggravate the stigma by labelling children of affected families as ‘at-risk’” [3].

Again, to grasp the potential risks, researchers need to learn about the local social and cultural context, and how affected individuals perceive potential risks. In this case, engagement with community members helped to identify a potential problem with secondary stigma. Consequently, researchers may need to protect family members by avoiding the identification of households whose members are research participants. As Case 5 illustrates, researchers may also need to consider how to protect participants from family members who might react poorly to research participation or publicizing a diagnosis. Finally, there is a question of whether other parties should be asked for consent, in addition to the participants themselves. Tekola et al. write: “Most participants said that patients are usually free to make their own decisions about participation in research. However, in relation to genetic research on podoconiosis, most participants suggested involving the head of the family, or the family as a whole in the consent process. Because of the prevailing stigma attached to a podoconiosis-affected family, they (by implication) preferred that the ownership of every sample for genetic study should belong to the whole family [3].”

Whether and how family members, or other third parties who might be affected by research, should be involved in decisions about research participation remains under-explored in research ethics.

## Conclusions

Research with stigmatized groups or on stigmatized conditions can pose substantial ethical challenges. That is a reason to conduct the research thoughtfully; it is not a reason against conducting the research at all. In thinking about research with stigmatized populations, researchers, funders, and RECs should avoid overprotecting these populations, whether by excluding them altogether, by instituting excessive protections, or by refusing to engage with controversial questions.

That said, research with stigmatized groups or on stigmatized conditions can entail different and greater risks than other areas of health research. Investigators and RECs need to be particularly attentive to these risks and how to manage them. A first step is for researchers to reflect on stigma and to identify their own prejudices that might affect their research. Second, researchers should be proactive in identifying potential risks and strategies to mitigate them. In doing so, they should think through each stage of the research—from the research questions, to recruitment methods, study visits, research procedures, and dissemination of results. Third, risks at all of these stages should be considered at the planning stage. For example, the dissemination plan should be made before the research starts, not once data is already in – even if adjustments have to be made along the way. Fourth, researchers should be aware that there may be additional or greater risks to stigmatized groups and so a more exhaustive analysis may be valuable (for example, risks of being seen visiting the clinic, risks of research procedures exacerbating stigma, risks to family members). Again, this does not mean that the research should not take place, nor that excessive protections should be instituted against risks that, when carefully assessed, turn out to be highly speculative. Fifth, engagement with affected individuals and communities is vital for the identification and mitigation of risk. The extensive literature on community engagement and good community participatory practice is a valuable resource for researchers working with stigmatized groups.

This analysis identified several outstanding challenges for the ethical conduct of research with stigmatized groups or on stigmatized conditions. Among these challenges are: (1) whether and when it might be acceptable to develop interventions to reduce unhealthy behaviors by de-normalizing them, at the risk of stigmatizing individuals who engage in those behaviors; (2) how, if at all, researchers should access the most severely stigmatized populations, when the stigma is a significant barrier to recruitment and the risks posed by research that might inadvertently signal the status of participants can be very high; and (3) best practices for minimizing risks to third parties, especially when dealing with populations at high risk for secondary stigma. Solutions to these outstanding ethical challenges are likely to be developed on a case-by-case basis so that they can be responsive to context-specific factors. Nonetheless, as we hope we have shown, even context-specific solutions can provide generalizable lessons from which others in the stigma research community can learn.

## Abbreviations

FAS: fetal alcohol syndrome
FASD: fetal alcohol spectrum disorders
MAO: monoamine oxidase
PrEP: pre-exposure prophylaxis
REC: research ethics committee
